# Effectiveness and safety of pure acupuncture and moxibustion in the treatment of children with cerebral palsy: A protocol for systematic review and meta analysis

**DOI:** 10.1097/MD.0000000000023907

**Published:** 2021-01-29

**Authors:** Yani Tang, Zhiliang Cao, Yun Xia, Yinghan Liu, Wei Zhang

**Affiliations:** The First Affiliated Hospital of Hunan University of Chinese Medicine, Changsha, Hunan province, China.

**Keywords:** acupuncture and moxibustion, children with cerebral palsy, protocol, systematically evaluate

## Abstract

**Background:**

Infantile cerebral palsy refers to brain damage in infants and young children during their development, causing brain dysfunction, mainly manifested as dyskinesia, which may be complicated by mental retardation, epilepsy, and bone and joint developmental disorders. Clinical practice shows that acupuncture can effectively treat children with cerebral palsy, but it needs to be proven. This research will systematically evaluate the clinical effectiveness and safety of acupuncture and moxibustion in the treatment of children with cerebral palsy, and provide evidence-based evidence for it.

**Method:**

Search the following databases, including CNKI, WANFANG, China Biomedical Database, VIP, PubMed, Embase, the Cochrane Library, Web of Science. The retrieval time is from the establishment of the databases to October 2020, collecting all clinical randomized controlled studies of acupuncture and moxibustion treatment of children with cerebral palsy. Two investigators independently extract and evaluate the data of the included studies, and use RevMan V.5.3 software to conduct meta-analysis of the included literature.

**Result:**

This study evaluates the effectiveness and safety of acupuncture and moxibustion in the treatment of children with cerebral palsy through indicators such as Gross Motor Function Measure Scale, the Modified Ashworth Scale, and so on.

**Conclusion:**

This study will provide reliable evidence-based evidence for the clinical application of acupuncture and moxibustion in the treatment of children with cerebral palsy.

**Ethics and dissemination:**

Private information from individuals will not be published. This systematic review also does not involve endangering participant rights. Ethical approval was not required. The results may be published in a peer-reviewed journal or disseminated at relevant conferences.

**OSF Registration number:**

DOI 10.17605/OSF.IO/7GUF5

## 1 Introduction

Cerebral palsy in children is a syndrome of non-progressive brain injury or developmental defects from conception to infancy, mainly manifested as dyskinesia, postural dysfunction and mental retardation, etc.^[1]^ The incidence of pediatric cerebral palsy in developed countries is about 1.5% to 2.5%, which is the main factor leading to pediatric disability and even death,^[2]^ which will have a serious impact on the quality of lives of patients, and also cause heavy economic burden on the family and society. Modern medical theory believes that the cause of cerebral palsy is complex and there are many risk factors. Genetic inheritance,^[3]^ congenital malformation, infection, premature birth, multiple pregnancy, placenta abnormalities, and neonatal encephalopathy will all increase the risk of cerebral palsy,^[4]^ but the pathogenesis of cerebral palsy is unclear. Western medicine clinics currently mostly adopt early intervention, rehabilitation and neurotrophic drug therapy^[5]^ for treatment,^[6]^ but the efficacy is poor and needs further evaluation and testing. Traditional Chinese medicine for the treatment of children with cerebral palsy mostly uses acupuncture, Tuina, traditional Chinese medicine, acupoint embedding and other therapies or combined therapy.^[7]^

Acupuncture is a traditional external treatment method that originated in China. It mainly uses filiform needle in acupuncture to stimulate specific acupoints. Traditional medicine believes that filiform acupuncture can regulate the circulation of qi and blood and promote the recovery of body functions.^[8]^ Clinical studies claim that acupuncture can effectively treat children with cerebral palsy^[9]^ and restore brain function.^[10]^ Acupuncture is simple, safe and effective, with few adverse reactions, and has great advantages in clinical treatment.

At present, although a large number of clinical research results show that acupuncture and moxibustion are effective in treating children with cerebral palsy, there are still some shortcomings. For instance, there are certain differences in the methodological design of each trial, some factors that interfere with the research results cannot be ruled out, and the results of a single study may not be applicable for the general population. Therefore, we have carried out a meta-analysis to include more research objects, systematically and comprehensively study all the literature on the treatment of children with cerebral palsy by acupuncture alone, and evaluate the effectiveness and safety of acupuncture alone in the treatment of children with cerebral palsy from an evidence-based perspective.

## 2 Method

### 2.1 Protocol register

This protocol of systematic review and meta-analysis has been drafted under the guidance of the preferred reporting items for systematic reviews and meta-analyses (PRISMA). In addition, it has been registered on open science framework (OSF) on November 26, 2020. (Registration number: DOI 10.17605/OSF.IO/7GUF5)

### 2.2 Ethics

This study only searches and evaluates existing literature resources. And it does not need to recruit patients, collect personal information, and require the approval of the ethics committee.

### 2.3 Eligibility criteria

#### 2.3.1 Types of studies

According to the guidelines of the Cochrane Collaboration, we will include randomized controlled trials on acupuncture and moxibustion in the treatment of children with cerebral palsy that meet the search criteria, without limitations of date, language and experimental design. And we will exclude animal studies and literature reviews.

#### 2.3.2 Research objects

Through Gross Motor Function Measure, video gait analysis, head imaging examination and other standard diagnosis,^[11]^ patients who are diagnosed as children with cerebral palsy are not limited by nationality, race, gender, course of disease, and location of disease.

#### 2.3.3 Interventions

Treatment group: pure acupuncture treatment

Control group: other non-acupuncture therapy or placebo treatment

#### 2.3.4 Outcome indicators

(1) Primary outcome: Gross Motor Function Measure Scale, the Modified Ashworth Scale.
(2) Secondary outcome: Activity of Daily Living Scales; Gesell Growth Table; Peabody development motor scale, Adverse Effects Rate.

### 2.4 Exclusion criteria

(1) Research literature belongs to the research whose original data cannot be obtained, such as abstracts and conference papers;
(2) Repeatedly published research, or different research repeated by research subjects;
(3) Research using non-randomized controls or wrong methodology;
(4) Research that has obvious logic error or incomplete data, which cannot be processed after contacting the author;
(5) The experimental group adopts non-acupuncture treatment or acupuncture combined with other therapies;
(6) The included research objects do not belong to the category of children with cerebral palsy.

### 2.5 Search strategy

Use infantile cerebral palsy (Xiao Er Nao Tan), acupuncture and moxibustion (Zhen Jiu), acupuncture (Zhen Ci), acupuncture treatment (Zhen Jiu Zhi Liao), etc. as search terms in CNKI, VIP, China Biomedical Database, WANFANG, PubMed, EMBASE, the Cochrane Library, Web of Science, and manually search related documents on Baidu Academic and Google Academic for supplement. The retrieval time is from the establishment of the databases to October 2020, and all the Chinese and English literatures on the treatment of children with cerebral palsy are collected. Take the retrieval strategy in PubMed as an example, as shown in Table 1.

**Table 1:** Search strategy in PubMed database.

Number	Search terms
#1	Cerebral palsy[MeSH]
#2	Cerebral palsy[Title/Abstract]
#3	CP[Title/Abstract]
#4	Cerebral Palsy, Dystonic-Rigid[Title/Abstract]
#5	Mixed Cerebral Palsies[Title/Abstract]
#6	Little disease[Title/Abstract]
#7	Spastic Diplegia[Title/Abstract]
#8	Athetoid Cerebral Palsy[Title/Abstract]
#9	Hypotonic Cerebral Palsy[Title/Abstract]
#10	Spastic Cerebral Palsy[Title/Abstract]
#11	#1 OR #2 OR #3 OR #4 OR #5 OR #6 OR #7 OR #8 OR #9 OR #10
#12	Acupuncture[Mesh]
#13	Acupuncture[Title/Abstract]
#14	Acupuncture Treatment[Title/Abstract]
#15	Pharmacoacupuncture[Title/Abstract]
#16	Pharmacoacupuncture Treatment[Title/Abstract]
#17	#12 OR #13 OR #14 OR #15 OR #16
#18	#11 And #17

### 2.6 Data screening and extraction

**Figure 1:**
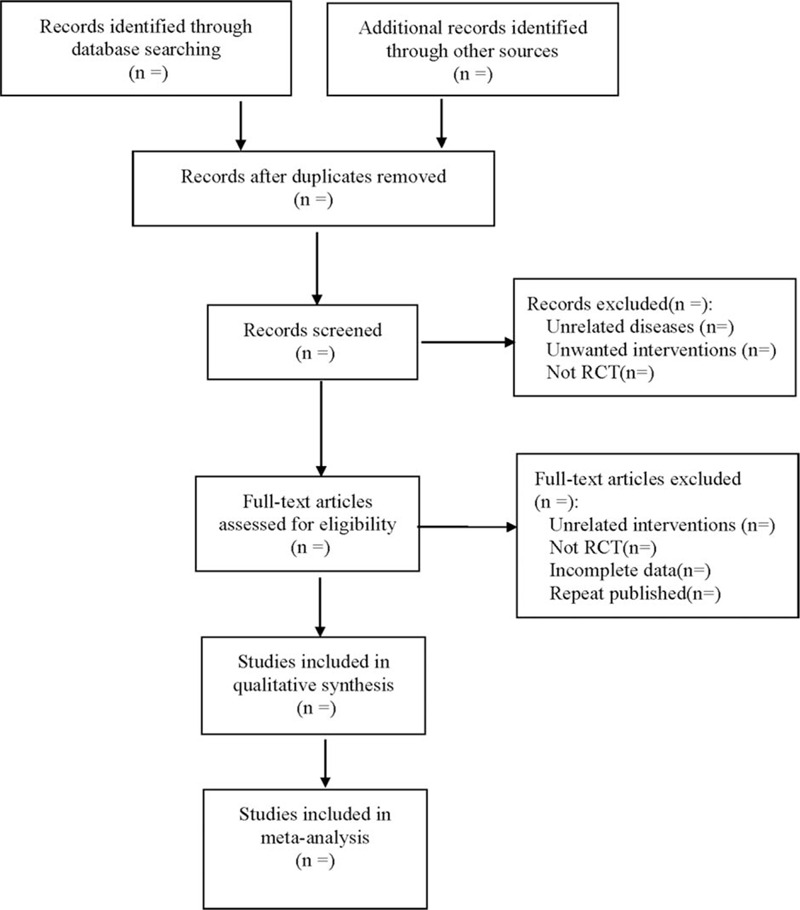
The process of literature screening.

We will use Endnote X9 software to manage all the studies that meet the search criteria, and strictly refer to the established inclusion and exclusion criteria to screen out eligible studies. The process will be completed independently by 2 researchers, and then cross-compared their results. If the conclusions of the 2 researchers are inconsistent, the differences can be resolved through discussion. If no agreement can be reached, it will be decided with the assistance of the third author. We will use the PRISMA flowchart to represent the selection process for the study. The flowchart is shown in Figure 1.

The 2 researchers will use the Microsoft Excel data extraction table to independently extract the data in the study. If the extraction results diverge, they will discuss. If an agreement cannot be reached, the decision will be made after consultation with third-party researchers. We will extract the following data:

(1) Research characteristics (author, title, year, random method, etc);
(2) Participants (sample size, gender, age, etc);
(3) Intervention (acupuncture selection of points, control treatment methods, etc);
(4) Control (treatment course, acupuncture technique, acupuncture time, etc);
(5) Outcome indicators (results of each indicator, adverse events and incidence, etc).

### 2.7 Literature quality evaluation

The risk of bias assessment tool in the Cochrane Handbook will be used to assess the methodological quality of randomized controlled trials. Each randomized controlled experiment is evaluated with reference to the following items: Random sequence generation, allocation hiding, blindness of the result data, completeness of the result data, selective reporting and other sources of bias are classified as low risk, high risk or unclear according to the evaluation results.

### 2.8 Statistical analysis

#### 2.8.1 Data analysis and processing

Two researchers use Stata15.0 to synthesize and analyze the valid data. Binary data will use risk ratio or odds ratio with 95% confidence interval, and continuous data will use a weighted mean difference with 95% confidence interval or standard mean difference. We will use the results of *I*^*2*^ test and *P* test as the criteria for judging heterogeneity. If the heterogeneity is considered to be low (*I*^*2*^ ≤ 50%, *P* ≥ .1), the fixed-effects model will be used to synthesize the data. If the heterogeneity is considered moderate (*I*^*2*^ > 50%, *P* < .1), the random effects model will be used. If the heterogeneity is high, a subgroup analysis or meta regression will be performed to reasonably explain and evaluate the potential source of heterogeneity. If meta-analysis is not feasible, a descriptive analysis will be performed.

#### 2.8.2 Dealing with missing data

We will contact the author to obtain missing or incomplete data. If the relevant data of the study cannot be obtained, we will abandon the meta-analysis.

#### 2.8.3 Subgroup analysis

Perform subgroup analysis for pure acupuncture and other therapies based on intervention methods; Perform subgroup analysis based on the duration of the intervention; Perform subgroup analysis according to different treatment courses; Perform subgroup analysis based on the acupuncture points used; According to the clinical manifestations, it can be divided into subgroups such as spasticity, involuntary movement, rigidity, and ataxia for analysis.

#### 2.8.4 Sensitivity analysis

We will conduct a sensitivity analysis under the influence of variables such as sample size, methodological quality, and missing data to check the stability and reliability of the combined results.

#### 2.8.5 Assessment of reporting biases

We will use a funnel chart to test the publication bias. If the symmetry is good, there is no obvious publication bias and the conclusion is highly reliable.

#### 2.8.6 Evidence quality evaluation

We will use the Grading of Recommendations Assessment, Development, and Evaluation) to assess the results. There are 5 factors that reduce the quality of evidence: Risk of bias, inconsistency, indirectness, imprecision and publication bias. There are 3 factors that can improve the quality of evidence: possible remaining confounding factors, dose-response gradient dose-effect evidence, and larger effect size. The quality of the evidence will be divided into 4 levels based on the above evaluation: very low, low, medium and high.

## 3 Discussion

Cerebral palsy in children belongs to the categories of 5 kinds of retardation, 5 weaknesses, flaccidity syndrome and dementia in traditional Chinese medicine.^[12]^ Traditional medicine believes that the etiology is mainly divided into congenital and acquired factors. The congenital factors are mostly related to insufficient endowment, infection or premature delivery. Acquired factors include improper care, improper care during pregnancy, birth injury, asphyxia, and neonatal infection with evil diseases.^[13]^ The pathogenesis is mainly due to insufficient qi, blood, yin and yang, or stasis of the pathological product, which leads to dysfunction of the viscera and various functions of the body.^[14]^

Acupuncture is 1 of the main intervention methods of traditional Chinese medicine. By stimulating the areas with rich nerve and vascular tissue, it can increase the body's metabolic level, produce nutrition for related tissues, and have the functions of regulating qi and blood and balancing yin and yang.^[15]^ Nowadays, acupuncture therapy is gradually standardized and systematized. In the process of continuous research and development, many systemic acupuncture programs for the treatment of cerebral palsy in children have been developed clinically.^[16]^ For example, scalp acupuncture mainly stimulates the corresponding area of the head according to the part of the brain and cooperates with the stimulation of the distal spinal cord.^[17]^ Modern medical research has found that acupuncture has great effect on the recovery of the nervous system and musculoskeletal system,^[18]^ and it has great advantages in improving motor dysfunction and repairing damaged brain cells in children with cerebral palsy.^[19]^

At present, although there are many reports on acupuncture treatment of children with cerebral palsy, there is a lack of evidence-based systematic reviews. Hence it becomes necessary to conduct an objective evaluation of evidence-based medicine for the treatment of acupuncture and moxibustion in children with cerebral palsy, promote acupuncture and moxibustion therapy, and provide scientific treatment prescriptions for the clinic. However, this study does have certain limitations. There is a lack of randomized controlled trials with large sample size and high quality. The diagnostic criteria and efficacy criteria for children with cerebral palsy are inconsistent. It is difficult to maintain complete follow-up information due to the particularity of the test subjects. There may be some methodological and clinical heterogeneity. Since the search language is limited to English and Chinese, there may be a certain publication bias.

## Author contributions

**Data collection:** Yani Tang and Yun Xia

**Funding support:** Wei Zhang

**Literature retrieval:** Yun Xia and Yinghan Liu

**Software operating:** Yun Xia and Yinghan Liu

**Supervision:** Zhiliang Cao

**Writing – original draft:** Yani Tang and Zhiliang Cao

**Writing – review & editing:** Yani Tang and Wei Zhang

## Abbreviations

Abbreviations: CI = confidence interval, GMFM = gross motor function measure, GRADE = The grading of recommendations assessment, development, and evaluation, OR = odds ratio, RCTs = randomized controlled trails, RR = risk ratio, SMD = standardized mean difference, WMD = weighted mean difference.
